# Toward New Paradigms in the Follow Up of Adult Patients With Celiac Disease on a Gluten-Free Diet

**DOI:** 10.3389/fnut.2019.00153

**Published:** 2019-10-01

**Authors:** Maria I. Pinto-Sanchez, Julio C. Bai

**Affiliations:** ^1^Department of Medicine, Gastroenterology Division, McMaster University Medical Center, Hamilton, ON, Canada; ^2^Farncombe Family Digestive Health Research Institute, Hamilton, ON, Canada; ^3^Research Institute, Universidad del Salvador, Buenos Aires, Argentina; ^4^Hospital de Gastroenterologia Dr. C. Bonorino Udaondo, Buenos Aires, Argentina

**Keywords:** celiac disease, follow-up, antibodies, gluten-free diet, biopsy

## Abstract

Gluten free diet is the only available treatment for celiac disease (CeD). Patients with CeD who do not adhere to a strict gluten-free diet (GFD) have been found to have complications involving nutritional deficiencies, increased risk of bone fractures, increased risk of mortality, and certain types of cancers. Complete removal of gluten from the diet in a patient with CeD often results in symptomatic, serologic, and histologic remission. However, strict compliance with the diet is challenging. Long-term follow-up care is needed to assure treatment compliance and positive health outcomes. Monitoring celiac specific serology, nutrient deficiencies, bone mineral density, and assessment of GFD compliance have been recommended in clinical practice. However, there is no consensus on which specific tests and how often they should be performed during the follow up. Here, we have performed a review of the literature on current strategies to follow up patients with CeD. There are new tools for monitoring adherence to the GFD which could change some paradigms in following up treated patients.

## Backcround

Celiac disease (CeD) is a chronic systemic, immune-mediated condition precipitated by exposure to dietary gluten in genetically pre-disposed individuals (1). It is a relatively common disorder which affects around 1% of the population worldwide, and the prevalence has been increasing in the last years (2–4). The hallmark of CeD is enteropathy immune mediated, with characteristic villous atrophy in the proximal small intestine. CeD often presents with malabsorptive symptoms, including diarrhea and weight loss; with non-specific symptoms, such as abdominal pain, anemia, or osteopenia; or may be completely asymptomatic (3). Independently on the type of presentation, untreated, or partially treated celiac disease is associated with persistent symptoms and complications including nutritional deficiencies, osteoporosis, infertility, increased malignancies, and increased mortality (5).

The only available therapy for CeD is a strict, lifelong, gluten-free diet (GFD), which requires the complete removal of all wheat (gluten), rye (secalin), and barley (hordein) products. It is known that 50 mg of gluten (6–8), which could be found in a few crumbs of bread or a small piece of pasta, can perpetuate the enteropathy in patients with CeD. Due to accidental or intentional gluten exposure (contamination with gluten), it is not possible for some people to remain totally gluten-free. Therefore, most of patients with CeD are restricted gluten diet rather than gluten-free. Clinical studies using methods for indirect assessment of GFD compliance, such as food interviews, dietary self-report, or follow-up serology showed that 17–80% of patients with CeD are not compliant with the GFD (9). Not surprisingly, their symptoms persist, and their small bowel does not heal (10). The negative psycho-social aspects of diet that is highly restricted, the need of permanent vigilance to avoid gluten, and the high frequency of inadvertent gluten exposure lead to low patient satisfaction and significant disease burden (11, 12). Patients with CeD often report decreased health-related quality of life (13) and a high treatment burden compared to those with other chronic diseases, including inflammatory bowel disease and type 1 diabetes; which are often perceived as more severe than celiac disease. For this reason, ideally immediately after diagnosis, patients with CeD should receive dietary counseling by an expert dietician in celiac disease and GFD compliance monitored in the follow up.

### Assessment of Disease Activity After the Diagnosis

Celiac disease (CeD) is a systemic inflammatory condition, and may lead to serious complications if not adequately controlled. Even though it has been recommended that patients with CeD visit regularly the clinic, and specific markers of celiac disease are monitored after the diagnosis (14–19); patients with CeD are not followed up consistently (20). Improving understanding of the role of symptoms and tests in the follow-up of patients with CeD could positively impact on disease management.

#### Role of Symptoms/Signs in the Follow Up of CeD Patients

**Figure 1:**
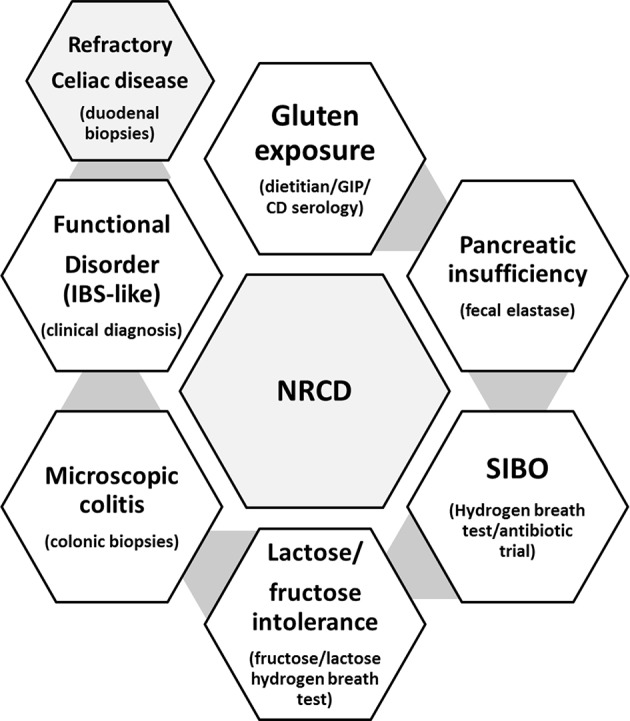
Common causes for persistent symptoms in the follow-up of patients with CeD (non-responsive celiac disease; NRCD), and common tests used in clinical practice for the diagnosis of each concomitant condition.

A substantial proportion of patients with CeD (~30%) have recurrent or persistent symptoms despite being on a GFD (21), and the most common cause are continued or intermittent, purposeful or inadvertent gluten ingestion (20). Other causes of non-responsive celiac disease could be related to exocrine pancreatic insufficiency, bacterial overgrowth, microscopic colitis, carbohydrates (fructose/lactose) intolerance, or functional disorders (16, 21) (Figure 1). However, symptoms are not always present to alert for gluten ingestion, and some patients with CeD may persist with enteropathy for years without been aware (22). Independently of the presence or absence of symptoms, celiac patients with CeD with persistent enteropathy are at increased risk of complications, including lymphoproliferative malignancy, compared to those with mucosal healing (HR 2.26; 95% CI, 1.18–4.34) (22, 23). To prevent complications, current guidelines recommend regular follow up and monitoring of GFD compliance in both symptomatic and asymptomatic patients with CeD. There is general agreement among guidelines (16) that patients with CeD should be monitored at least two times in the first year after diagnosis, to assess disease activity, nutrition, dietary adherence, and bone health status (Table 1).

**Table 1:** Comparison of guidelines recommendations for follow up of adult patients with CeD.

Assessment^*^	**ACG (16)**	**BSG (18)**	**WGO (19)**	**Kelly et al. (14)**	**AGA (15)**	**ESsCD (17)**
**Clinical** Short term Long term	Every 6 months Annually	No specific recommendations	Annually after the 1st year in adults	Annually or if recurrent symptoms	No specific recommendations	3–4 months Annually
**CeD serology** Short term Long term	Every 6 months Annually	1 year	tTG IgA or DGP IgA Every 3–6 months until normal, then/1–2 years	Serology every 3–6 months until normal, then/1–2 years	Every 6 month Annually	tTG IgA 3–4 months Annually
**Duodenal biopsy** Short term Long term	If persistent Reasonable 1–2 year^**^	Not mandatory if asymptomatic	In symptomatic seronegatives at follow-up. Unclear in asymptomatic	Not mandatory. Consider 1–2 year. after diagnosis	If symptomatic If persistent enteropathy	If symptomatic Reasonable 1–2 year^**^
**Screening for autoimmune** Short term Long term	No specific recommendations	No specific recommendations	No specific recommendations	At diagnosis, then ev 1–2 year	No specific recommendations	At diagnosis E. 1–2 year
**BMD** Short term Long term	unclear unclear	In high risk for osteoporosis. Repeat if abnormal.	Baseline. Repeat if abnormal or at meno-andropause if normal	Baseline. Repeat if abnormal	No specific recommendations	Baseline If abnormal
**GFD compliance** Short term Long term	Every 3–6 month Annually	No specific recommendations	Every 3–6 months until normal, then/1–2 years. Potential use of GIP	No specific recommendations	No specific recommendations	3–4 month E. 1–2 year
**Nutritional** Short term Long term	Every 3 months until normal Annually	No specific recommendations	Every 3–6 months until normal, then/1–2 years	Every 3–6 months until normal, then/1–2 years	No specific recommendations	Baseline Annually
**Vaccine**	No specific recommendations	Pneumococci, in Hyposplenism*H. influenzae*Unclear	Pneumococci, H. influenzae, and meningococci should be performed	No specific recommendations	No specific recommendations	Pneumococci, in Hyposplenism *H. influenzae* Unclear

* *Short term, <2 year after diagnosis; long-term, >2 year after diagnosis; DGP, Deaminated gliadin peptides*.

** *It may be reasonable to do a follow-up biopsy in adults after 1–2 years of starting a GFD to assess for mucosal healing, especially in patients older than 40 years or in those having initially severe presentations*.

#### CeD Specific Serology in the Follow Up

IgA antibodies to TG2 and to deamidated gliadin peptides (DGPs) are commonly used to monitor celiac disease activity in the follow up (24). Although it takes several months for CeD specific serology to become under the normal cutoff level, a significant decrease in serology levels over the first year is suggestive of GFD adherence, and patients with CeD whose serologic features do not improve should be re-assessed for gluten exposure (25). However, negativity of CeD specific serology does not reflect strict compliance with GFD. In adult patients with CeD on a GFD, CeD serology is poor predictor of dietary transgressions (26). Although the CeD antibody tests show a high accuracy for the diagnosis of CeD, these tests are not as reliable in the follow up as they don't correlate well with histological findings or symptoms either (23). However, it is important to highlight that a negative CeD specific serology in a treated patient, does not necessarily guarantee intestinal mucosal healing (23, 26).

Even though CeD specific serology is imperfect test, guidelines recommend to assess CeD serology (anti- tissue transglutaminase; tTG IgA, or DGP IgA) every 3–6 months in the first year after the diagnosis or until stabilization, and then annually in the long term to monitor CeD activity (16). In cases of IgA deficiency, DGP IgG, and tTG IgG are recommended (14–16, 19).

#### Role of Endoscopy in the Follow Up

Repeated endoscopy with duodenal biopsies in the follow up has been controverted. There is currently no evidence indicating that performing routine follow-up biopsy is needed for all patients with CeD (17). Endoscopy is expensive, relatively invasive, and impractical procedure for regular disease activity monitoring. It has been suggested that biopsies should be repeated in the follow up of patients with CeD 2 years after the diagnosis to confirm mucosal healing (5, 15–17, 23). However, others have discouraged this practice based on previous demonstration of persistent damage in adults for years despite strict compliance with GFD (27, 28). There is general consensus that patients with persistent or newly developed symptoms without clear explanation, should undergo endoscopic biopsies to assess mucosal healing even if TG2-IgA levels are within normal range (23). Even though mucosal healing is likely in asymptomatic patients with negative serology on a GFD, studies suggested increased risk of lymphoma and mortality in this population with persistent inflammation (27). Therefore, current guidelines find reasonable a follow-up biopsy after 1–2 years of GFD, with the idea to assess mucosal healing, especially in patients over the age of 40 years or in those with severe presentations (16, 28). However, these recommendations are based on expert advice, and evidence on benefit of this strategy on long term outcomes is still lacking.

#### Nutritional Deficiencies in the Follow Up

Nutritional deficiencies in CeD may be directly related to celiac enteropathy, or could develop as a consequence of nutrients restriction associated to the GFD; or a combination of both factors (16, 29). The most common micronutrient deficiency is iron; however, iron stores typically improve on a GFD. Iron supplementation may be needed in a subset of patients with CeD. Folate, vitamin B12, vitamin D, and zinc are commonly deficient in patients with CeD in the follow up and often require supplementation (16). Table 2 denotes the most frequent micronutrient deficiencies in celiac disease, and suggested supplementation.

**Table 2:** Common nutrient deficiencies in the follow up of adult patients with CeD and recommended oral supplementation.

**Nutrient**	**Supplementation dose**	**Comments**
Iron	*Oral supplements* Ferrous gluconate: 300 mg (35 mg) 1–3 tab. bid to tid Ferrous fumarate: 300 mg (100 mg) 1 tab. bid Ferrous sulfate : 300 mg (60 mg) 1 tab tid Heme Iron : 398 mg (11 mg Heme) 1 tab tid Polysaccharide: 150 mg (150 mg) 1 caps. Daily IV iron Iron sucrose: 200–300 mg 3–5 doses Iron dextran: 510 mg weekly × 2 doses	^*^Iron and ferritin at diagnosis ^*^Vitamin C (500 units) may increase iron absorption ^*^Zinc decrease absorption ^*^IV iron should be considered in severe cases or intolerance to oral supplementation
Vitamin D	1,000–2,000 IU/day	^*^Taken with calcium to increase absorption
Folate	400–800 mcg/day	^*^Increased needs in pregnancy
B12	1,000–1,200 mcg/day	^*^Sublingual formulation available
Zinc	25–50 mg/day	^*^High zinc supplementation may lead to copper deficiency
Copper	2–4 mg/day	^*^Zinc and iron decrease copper absorption
Calcium	1,000–1,500 mg/day	^*^taken with vitamin D to increase absorption
Fiber	25–30 g/day	^*^Psyllium and Inulin most common encourage fluids
Chromium	200 mcg/day	^*^Interaction with PPIs, NSAIDS, and levothyroxine

* *Testing for nutrients is recommended at diagnosis and if abnormal, repeat every 3–6 months until normal. Then once every 1–2 years*.

It is strongly recommended that patients with CeD is assessed by an expert dietitian, to provide education on GFD and develop dietary strategies to help with symptoms management (16, 29).

#### Bones Disease in the Follow Up

Bone health can be negatively affected in CeD owing to the inflammatory process and malabsorption of calcium and vitamin D (30, 31). Osteopenia and osteoporosis and bone fractures are the most common complications associated with celiac disease (32). The risk of bone fractures is increased in celiac disease (33) regardless of the presence of symptoms; and the excess risk is reduced with adherence to GFD (34).

Testing of BMD should be performed at diagnosis of celiac disease before deciding on further management (35). In those with osteoporosis or osteopenia at diagnosis or those who do not adhere to a GFD, a follow up BMD after at least 1 year of supplementation with calcium and vitamin D is recommended (31).

In addition to ensure strict GFD, it is prudent to ensure adequate calcium and vitamin D intake for all patients with CeD. If after 1–2 years of adhering to a GFD and including appropriate calcium and vitamin D supplementation the patient continues to show signs of osteoporosis, the addition of specific osteoactive treatments should be considered (31); despite no clear evidence on the magnitude of the benefit compared to the strict GFD alone. A recent study (30) has shown that a strict GFD improves the microstructural parameters of the bones, which is often difficult to reach, even with osteoactive treatment.

#### Monitoring Thyroid Function in the Follow Up

Celiac disease (CeD) has been associated to other autoimmune conditions, being the most frequent type 1 diabetes and autoimmune thyroiditis (36).

Autoimmune thyroid disease, especially Hashimoto's hypothyroidism is more frequent in patients with CeD (37). However, we need to consider that low-titer false-positive anti-tTG may occur in patients with thyroid disease (19).

There has been discussion on whether a gluten-free diet in CeD protects against thyroid disease or modifies the natural history of the disease. At least two studies (38, 39) suggest that gluten-free diet compliance does not influence on the development of thyroid disease. Regardless of the degree of compliance with the diet, experts recommend to monitor for thyroid disease in the follow up of patients with CeD (40). How frequent the thyroid tests should be ordered in the follow up of patients with CeD is not clearly stated.

### Challenges of Monitoring of GFD Compliance

The management and follow-up of patients with CeD is preferentially performed with a team-based approach in which the dietician has an important role (15, 16) in the practical advice on lifestyle and choice of foods. It is well-known that 50 mg of gluten, which is equivalent to a few crumbs of bread or pasta, can produce symptoms and/or increase intestinal inflammation in patients with asymptomatic CeD; therefore, maintaining a lifelong GFD is necessary for all patients (25). The compliance with the diet could be impaired either with inadvertent or purposely gluten intake. Inadvertent gluten intake could be due to lack of proper knowledge, or lack of control on contamination; for example, when eating outside home.

A dietary assessment by an expert dietitian, generally based on an interview or food diary/food frequency questionnaire, is considered an objective, non-invasive, and low-cost way to measure adherence to a GFD (15, 16). However, a detailed dietary review for assessment of compliance with the diet is time consuming (between 45 min and 1 h), expensive to the healthcare system and limited by the lack of expert dietitians. Therefore, due to limited resources, it is not commonly performed in the community; with consequent limitations in the management of patients with CeD. In addition, individuals are not very accurate when reporting their adherence level, and whether intentionally or not, dietary review may not identify involuntary infringements. Identifying immunogenic peptides (9) either in stool, urine, or in food is a promising new tool to assess inadvertent gluten ingestion when patients are not under control of preparing their meals.

#### Gluten Immunogenic Peptides

There is an increasing interest on the role of certain gluten immunogenic peptides (GIP), such as 33-mer, that are resistant to digestion and are recognized triggers of immune reaction in celiac disease. In their study, Comino et al. (9) described a relatively new method to monitor GFD adherence by detection of GIP in stool samples 6–48 h after any intake of gluten by using the G12 monoclonal antibody. GIPs are excreted in feces after gluten is ingested; therefore, detection in stools of patients with CeD on a GFD reflects gluten exposure. GIPs could be detected in stool after ingestion of as little as 50 mg of gluten (equivalent to a penne noodle). This amount is clinically relevant as estimated ingestion of that amount of gluten per day has been proven to induce mucosal damage in patients with CeD. The sensitivity and specificity of GIP testing in stool demonstrated in recent studies were 98.5 and 100%, which highlight the potential clinical usefulness of this new method as a marker of adherence to GFD in adults and children with CeD (41). Fecal GIP analysis has been proposed as a non-invasive and accurate method for a direct and quantitative assessment of gluten exposure. More recently, new tools for detection of GIP in stool and urine has been developed based on lateral flow immunoassays and the point-of-care technology. Based on these new tools, Costa et al. (41) have explored their utility for detecting GFD indiscretions in comparison with three-day dietary reports. The new tools for exploring GIP in stool is more sensitive than dietary reports in detecting short-term gluten exposure in patients with CeD on GFD, regardless of symptoms. Therefore, fecal GIP testing may help to guide patients with CeD during the treatment, as they often are exposed to gluten in the follow up, probably due to decreased awareness for cross contamination as the treatment progresses. These methods can complement the dietitian assessment of GFD compliance and clinical management of CeD.

### When Gluten Free Diet Is Not Sufficient: Non-responsive and Refractory Celiac Disease

A great proportion of patients during the follow up present symptoms despite adhering to the gluten free diet, and this is known as Non-Responsive Celiac Disease (NRCD) (21). The most common reason for NRCD is the persistent stimulation by gluten1 (5, 21). Dietitian assessment plays a key role in identifying sources of unaware contamination with gluten. In the case of strict compliance with the diet, other concomitant conditions including small intestinal bacterial overgrowth, pancreatic insufficiency, parasite infections, or functional disorders such as IBS-like symptoms should be investigated (13, 15). The presence of persistent enteropathy in duodenal biopsies after 1 year of strict gluten free diet may suggest a rare complication known as refractory celiac disease (RCD) (15, 42). Further investigations of immunohistochemistry, PCR and flow cytometry will help to differentiate between refractory type 1 and 2. This differentiation is important, as RCD Type 2 is associated with worse prognosis and increased rates of mortality (42).

A strict GFD should be encouraged and monitored in patients with NRCD and RCD. Additional therapies will be required to treat the concomitant condition leading to persistent symptoms in NRCD, such as courses of antibiotics for SIBO, pancreatic enzymes for pancreatic insufficiency or motility agents for IBS-like symptoms. For RCD, treatment with budesonide or other immunosuppressants will be needed to control inflammation, as well as repeated biopsies in the follow up and images to monitor the disease and rule out further complications (42, 43). Patients with RCD will benefit from the referral to a specialized center for further management of their condition (43, 44).

### What Are the Benefits of Following Up of Patients With CeD and Monitoring Their GFD Compliance?

Celiac disease is a chronic inflammatory condition, and persistence of inflammatory state may lead to complications including nutritional deficiencies, osteoporosis and increased risk of certain types of cancer (45). The risk for complications is increased in persistent active disease, regardless of the presence or absence of symptoms. It is well-known that a compliance with the GFD will lead to disease control in a great majority of patients with CeD, and consequently, decreased risk of complications and mortality (23). A strict gluten free diet is difficult to follow, and patients often are exposed to gluten in the follow up. Therefore, guidelines recommend adequate follow up to monitor for GFD compliance to prevent serious complications associated to the condition. Table 3 summarizes recommendations for follow up of patients with CeD.

**Table 3:** Recommended follow up for patients with CeD.

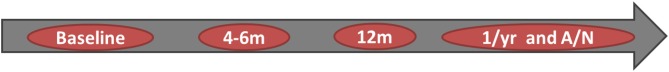			
✓ Wt,Ht	✓ Wt,Ht,	✓ Wt,Ht,	✓ Wt,Ht,
✓ PE	✓ PE	✓ PE	✓ PE
✓ Ed. GFD	✓ Ed. GFD	✓ Ed. GFD	✓ Ed.GFD
✓ RD	✓ RD (by request)	✓ RD (by request)	✓ RD (by request)
✓ CCA	✓ Serology	✓ Serology	✓ Serology
✓ Nutrients	✓ Lab (if abn)	✓ Lab (if abn)	✓ Lab (if abn)
✓ Serology			✓ BMD every 2 year (if abn)
✓ Liver			
✓ TSH			
✓ BMD			
^*^Offer GIP test			

* *RD, registered dietitian; wt ht, weight and height; PE, physical examination; Ed.GFD, education on gluten free diet; TSH, thyroid stimulant hormone levels; BMD, bone mineral density; Lab laboratory; if abn, if abnormal; 1/yr, once per year; A/N, as needed*.

### Who Should Follow Celiac Patients?

There is no consensus on whom and how should patients with CeD be followed-up, and there were several studies attempting to clarify this issue. Whether a great proportion of patients preferred to be followed-up by both a dietitian and a doctor (46) a study from Finland, demonstrated that follow-up by primary care providers is also effective (47). If experienced, primary care physicians should be responsible of following up patients with celiac disease.

## Conclusion

Patients with CeD should be monitored in the short and long term to ensure an adequate control on disease activity; regardless symptoms are present or not. Even though there is consensus on the need of clinical, serological, nutritional, and bone health status assessment in the follow up, there are still areas of uncertainty. The development of new tools will lead to changes in strategies to explore adherence to treatment in patients with CeD. Studies involving long term follow up are encouraged to clarify the role of endoscopy and of new tools to monitor GFD compliance on disease outcomes.

## Author Contributions

MP-S and JB designed the review, wrote the manuscript, and approved the final version.

### Conflict of Interest

The authors declare that the research was conducted in the absence of any commercial or financial relationships that could be construed as a potential conflict of interest.
